# A field efficacy trial of a trivalent vaccine containing porcine circovirus type 2a and 2b, and *Mycoplasma hyopneumoniae* in three herds

**DOI:** 10.1002/vms3.657

**Published:** 2021-10-22

**Authors:** Hyungmin Um, Siyeon Yang, Taehwan Oh, Hyejean Cho, Kee Hwan Park, Jeongmin Suh, Chanhee Chae

**Affiliations:** ^1^ College of Veterinary Medicine Department of Veterinary Pathology Seoul National University Gwanak‐gu Republic of Korea

**Keywords:** enzootic pneumonia, *Mycoplasma hyopneumoniae*, porcine circovirus type 2, porcine circovirus‐associated diseases, vaccine

## Abstract

**Background:**

This field trial was designed to evaluate the efficacy of a new trivalent vaccine containing porcine circovirus type 2a and 2b (PCV2a/b), and *Mycoplasma hyopneumoniae* at three independent locations.

**Methods:**

Three farms were selected based on their history of PCV2 and *M. hyopneumoniae* co‐infection. Each farm housed a total of 60, 3‐day‐old pigs that were randomly allocated to one of three treatment groups. Pigs were administered the trivalent vaccine intramuscularly with either a 1.0 ml dose at 3 and 24 days of age or a 2.0 ml dose at 21 days of age in accordance with the manufacturer's recommendations.

**Results:**

Clinically, the average daily weight gain of the one‐dose and two‐dose vaccinated groups within all three farms was significantly higher (*p* < 0.05) than those of unvaccinated animals during the growing (70–112 days of age), finishing (112–175 days of age) and overall (3–175 days of age) stages of production. One‐dose and two‐dose vaccinated animals elicited neutralizing antibodies and interferon‐γ‐secreting cells (IFN‐γ‐SC), which reduced the amount of PCV2 in terms of blood load and reduced the severity of lymphoid lesions when compared with unvaccinated animals. Similarly, one‐dose and two‐dose vaccinated animals elicited IFN‐γ‐SC, which reduced the amount of *M. hyopneumoniae* in terms of laryngeal load and reduced the severity of lung lesions.

**Conclusions:**

The intramuscular administration of either one or two doses of trivalent vaccine was not significantly different in any of the evaluated parameters. The results of field trial demonstrated that the trivalent vaccine was efficacious in the protection of swine herds where PCV2d and *M. hyopneumoniae* were in active circulation.

## Abstract 2

This field trial was designed to evaluate the efficacy of a new trivalent vaccine containing porcine circovirus type 2a and 2b (PCV2a/b), and Mycoplasma hyopneumoniae at three independent locations.

## 1 INTRODUCTION

Porcine circovirus type 2 (PCV2) is a very small, circular, single‐stranded DNA virus. It is a primary etiologic agent of ‘porcine circovirus–associated disease’ (PCVAD) (Afolabi et al., 2017; Chae, 2005). Since introduction of efficacious PCV2 vaccines, subclinical infection is currently the most common form of PCV2 infection worldwide (Segalés, 2012). The only observable disease manifestation associated with subclinical PCV2 infection is a decrease in average daily gain (ADG) (Alarcon, Rushton, Nathues, et al., 2013a; Alarcon, Rushton, & Wieland, 2013b; Kurmann et al., 2011). PCV2 is currently further divided into eight genotypes, designated as ‘a to h’ (Franzo & Segales, 2018). The second global genotype shift from PCV2b to PCV2d in 2014 (Xiao et al., 2015) marked the worldwide spread of PCV2d, launching it as the most prevalent PCV2 genotype in Asia and North America (Dinh et al., 2021; Franzo et al., 2016; Kwon et al., 2017; Thangthamniyom et al., 2017; Tsai et al., 2021; Yang et al., 2018).

*Mycoplasma hyopneumoniae* lacks a cell wall, has a very small amount of genetic material and is one of the smallest bacteria in nature (Razin et al., 1998). Enzootic pneumonia, caused by *M. hyopneumoniae*, is one of the most prevalent diseases affecting swine production and inflicts significant economic losses due to the resulting reduced growth rate and feed conversion efficiency (Young et al., 2011).

Co‐infection of PCV2 with *M. hyopneumoniae* caused major worldwide economic losses within the swine industry. Vaccination against PCV2 and *M. hyopneumoniae* is therefore routinely and widely used in the Asian pig industry. A new trivalent vaccine containing PCV2a and 2b (PCV2a/b) along with *M. hyopneumoniae* (registered as Fostera^®^ Gold PCV MH in the United States and Asia/CircoMax^®^ Myco in Europe, Zoetis, Parsippany, NJ, USA) has been introduced into the Asian market. The trivalent vaccine is of particular interest because it contains PCV2b, which is genetically close to PCV2d. Although PCV2a‐based vaccines may protect pigs against PCV2d (Opriessnig, Gerber, Xiao, Halbur, et al., 2014; Opriessnig, Gerber, Xiao, Mogler, et al., 2014; Opriessnig et al., 2017; Park et al., 2019), vaccine failure has also been reported in PCV2a‐vaccined herds (Opriessnig et al., 2013; Ramos et al., 2015; Seo, Park, Kang, et al., 2014). The objective of this study was to determine the efficacy in relation to growth performance of a new trivalent vaccine containing PCV2a/b and *M. hyopneumoniae* in pig farms suffering from concurrent circulation of PCV2d and *M. hyopneumoniae*.

## 2 MATERIALS AND METHODS

### 2.1 Farm history

The clinical field trial was conducted on three farms from June to December of 2020. Farms were labelled as ‘A, B and C’ and were 380‐, 260‐, and 430‐sow, respectively, farrow‐to‐finish swine operations with an all‐in‐all‐out production system. The status of porcine reproductive and respiratory syndrome virus (PRRSV) on all three farms was stable, with no active PRRSV circulation (high‐parity sows were the only seropositive animals in the herd). All replacement gilts used in the three farms tested seronegative for *M. hyopneumoniae* and were vaccinated for PCV2 on arrival. Sows from three farms were not immunized for either PCV2 or *M. hyopneumoniae*. All piglets received vaccinations for PCV2 and *M. hyopneumoniae* at 3 weeks of age, classical swine fever virus and *Erysipelothrix rhusiopathiae* at 6 weeks of age and foot and mouth disease virus at 8 and 12 weeks of age. Pigs were weaned at 21 days of age.

Each farm consistently suffered pig loss over several months due to growth retardation and respiratory disease in the late post‐weaning and growing stages. Clinical signs first appeared at approximately 7–10 weeks of age and reached peak mortality (approximately 1%–3% = farm A, 1%–2% = farm B and 2%–5% = farm C) between 10 and 15 weeks of age.

Farms A and B were selected based on their subclinical PCV2 infection and enzootic pneumonia. Previous diagnoses fulfilled the definition of subclinical PCV2 infection (Segalés, 2012) to include decreased ADG without overt clinical signs, absence of or minimal histopathological lesions in superficial inguinal lymph nodes and a low amount of PCV2 antigen presence in superficial inguinal lymph nodes as determined by immunohistochemistry in three out of five suspected pigs on the two farms. PCV2d was detected in serum from three pigs from each of these two farms, where log_10_ DNA copies/ml ranged from 2.35 to 3.23 from farm A and 2.45 to 3.32 from farm B. These values were consistent with the definition of subclinical PCV2 infection (Chae, 2004; Segalés et al., 2005). A lung examination was performed at the slaughterhouse, and was suggestive of enzootic pneumonia with cranioventral bronchopneumonia lesions in 60% of the 30 pigs had. Farm C was selected based on its clinical history of PCVAD and enzootic pneumonia. Previous diagnoses fulfilled the definition of PCVAD (Chae, 2004) to include clinical signs (i.e. retardation of growth), histopathological findings (i.e. lymphoid depletion and lymphoid granulomatous inflammation with intracytoplasmic inclusion bodies), along with PCV2 antigen presence in lymphoid lesions as determined by immunohistochemistry in four out of five suspected animals on the farm. PCV2d was detected in serum from three pigs that ranged from 4.35 to 5.18 log_10_ DNA copies/ml, which was consistent with defined PCVAD (Darwich et al., 2008; Segalés et al., 2005). A lung examination was performed at the slaughterhouse, which confirmed that 20% of the 30 pigs had mycoplasmal pneumonia lesions.

### 2.2 Study design

The results of this field study will be sent for registration and therefore strictly adhered to the guidelines of the Republic of Korea's Animal, Plant & Fisheries Quarantine & Inspection Agency (QIA, http://www.qia.go.kr). QIA protocols mandate that a total of 20 pigs were assigned to each study group. Study design considerations included randomization, personnel blinding and that animals were both weight‐matched and sex‐matched under a controlled clinical field trial format. To minimize sow variation, either six or nine, 3‐day‐old pigs were randomly selected from seven total sows. If six (or nine), 3‐day‐old pigs were pulled from a sow, two (or three) pigs were assigned to each of three uniform study groups. A total of 180 pigs were used for the entire study. Sixty pigs per farm were randomly divided into three groups within each farm (20 pigs per group; 10 = male and 10 = female) using the random number generator function (Excel, Microsoft Corporation, Redmond, WA, USA) (Table 1).

**TABLE 1:** Experimental design

Farm	Group	No. of pigs	Vaccination (dosage)
A	VacA1	20	D 18 (21 days of age; 2 ml)
	VacA2	20	D 0 (3 days of age; 1 ml), D 21 (24 days of age; 1 ml)
	UnVacA	20	None
B	VacB1	20	D 18 (21 days of age; 2 ml)
	VacB2	20	D 0 (3 days of age; 1 ml), D 21 (24 days of age; 1 ml)
	UnVacB	20	None
C	VacC1	20	D 18 (21 days of age; 2 ml)
	VacC2	20	D 0 (3 days of age; 1 ml), D 21 (24 days of age; 1 ml)
	UnVacC	20	None

The pigs in the VacA1, VacB1 and VacC1 groups were injected intramuscularly in the right side of the neck at study day 18 (21 days of age) with 2.0 ml of the trivalent vaccine containing PCV2a/b and *M. hyopneumoniae* (Fostera Gold PCV MH, Zoetis). Each farm received a different serial of the vaccine as follows: Farm A = Serial No. 395164A, Expiration date: 10 December 2021, Farm B = Serial No. 394687A, Expiration date: 10 December 2021 and Farm C = Serial No. 413369A, Expiration date: 03 February 2022. Pigs in the VacA2, VacB2 and VacC2 groups were injected intramuscularly in the right side of the neck at study days 0 (3 days of age) and 21 (24 days of age) with 1.0 ml of the trivalent vaccine. Pigs in the UnVacA, UnVacB and UnVacC groups were injected intramuscularly in the right side of the neck at study days 0 (3 days of age) and 21 (24 days of age) with 1.0 ml of phosphate‐buffered saline (PBS; 0.01 M, pH 7.4).

At 28 days of age, pigs from the vaccinated and unvaccinated groups were commingled and randomly assigned into six pens (10 pigs per pen) using the random number generator function (Excel, Microsoft Corporation). All pens were identical in design with equipment including free access to water and feed. Five pigs from each group were randomly selected and euthanized for necropsy at 112 days of age. The rest of pigs from each group were euthanized for necropsy at 175 days of age. Pigs were sedated by an intravenous injection of sodium pentobarbital and then euthanized by electrocution as previously described (Beaver et al., 2001). Lung, liver, tonsil, kidney, spleen, small and large intestine and superficial inguinal lymph node tissues were collected from each pig at the time of necropsy. Tissues were fixed for 24 h in 10% neutral buffered formalin, routinely processed and embedded in paraffin. The protocol for this field study was approved by the Seoul National University Institutional Animal Care and Use Committee (approval number SNU‐191017‐10).

### 2.3 Sampling collection

Blood and laryngeal swabs were collected at study days 0 (3 days of age), 18 (21 days of age), 46 (49 days of age), 67 (70 days of age) and 109 (112 days of age). Pigs were snared and restrained with a mouth gag for laryngeal swab collection. Swabs were guided with a laryngoscope down into the larynx. The internal walls of the laryngeal cartilages were then swept with the swabs once the larynx was visualized and the epiglottis was in a low position as previously described (Pieters et al., 2017).

### 2.4 Mortality

Pigs that died were subjected to gross pathological examination within 24 h at local veterinary practitioners. All major organs such as brain, lung, superficial inguinal lymph node, small and large intestine, liver, kidney and tonsils were collected from each pig submitted to the diagnostic laboratory. Polymerase chain reaction assays were used in order to detect specific nucleic acids for PCV2, PRRSV, swine influenza virus and *M. hyopneumoniae* (Cai et al., 2007; Chung et al., 2002; Kim & Chae, 2004a; Lee et al., 2008). All other bacterial isolation and identifications were carried out by using routine methods.

### 2.5 Clinical observations

Pig physical condition was monitored daily, and pigs were scored weekly for clinical signs as previously described (Seo, Park, Park, et al., 2014). Briefly, scoring was defined as follows: 0 (normal), 1 (rough haircoat), 2 (rough haircoat and dyspnoea), 4 (severe dyspnoea and abdominal breathing), 5 (severe dyspnoea and abdominal breathing, and hesitation of movement) and 6 (death). Scoring observers were blinded to vaccination status.

### 2.6 Growth performance

Pigs were weighed at study days 0 (3 days of age), 18 (21 days of age), 67 (70 days of age), 109 (112 days of age) and 172 (175 days of age). ADG (g/pig/day) was determined for study days 0–18, 18–67, 67–109 and 109–172. The ADG during these various production stages was calculated as the difference between the starting and final weight divided by the duration of the stage. Data for dead or removed pigs were included in the calculation.

### 2.7 PCV2 DNA in blood

A commercial kit (QIAamp DNA Mini Kit, QIAGEN, Valencia, CA, USA) was used to extract DNA from serum samples for PCV2d. The number of genomic DNA copies for PCV2a, PCV2b and PCV2d was then quantified by real‐time PCR (Gagnon et al., 2008; Jeong et al., 2015). To construct a standard curve, real‐time PCR was performed in quadruplicate in two different assays: (i) 10‐fold serial dilutions of the PCV2 plasmid were used as the standard, with concentrations ranging from 10^10^ to 10^2^ copies/ml, and (ii) 10‐fold serial dilutions of PCV2 cultured in PCV1‐free PK‐15 cells were used at concentrations ranging from 10^4.5^ TCID_50_/ml to 10^−3.5^ TCID_50_/ml. The PCV2 plasmid was prepared as described previously (Gagnon et al., 2008). Culture supernatants of PCV1‐free PK‐15 cells were used as negative control.

### 2.8 *Mycoplasma hyopneumoniae* DNA in laryngeal swabs

DNA was extracted from laryngeal swabs using the commercial kit (QIAamp DNA Mini Kit, QIAGEN, Valencia, CA, USA) to quantify the *M. hyopneumoniae* genomic DNA copy numbers by real‐time PCR as previously described (Dubosson et al., 2004). The forward and reverse primers (5′‐TTG ACT GCT ATC TTT GCA CGA TAA G‐3′ and 5′‐ ACA ATA ATT GCT GAC CGT GGC‐3′) and probe (5′‐FAM‐TGT CCA CTG CTG CAA ATA TTC GAT TTC TTG AA‐TAMRA‐3′) were used to detect *M. hyopneumoniae* (Dubosson et al., 2004).

To construct a standard curve, real‐time PCR was performed in quadruplicate in 10‐fold serial dilution of chromosomal DNA from *M. hyopneumoniae* strain SNU98703, with concentrations ranging from 10 ng/μl to 1 fg/μl. One femtogram of chromosomal DNA from *M. hyopneumoniae* is considered to be approximately one genome equivalent (Kurth et al., 2002). A positive and negative control was included in each run using chromosomal DNA from *M. hyopneumoniae* strain SNU98703 and double distilled water, respectively, as the template.

### 2.9 Serology

The presence of PCV2 and *M. hyopneumoniae* antibodies was evaluated in serum samples by use of commercially available enzyme‐linked immunosorbent assay [ELISA kits (SERELISA PCV2 Ab Mono Blocking, Synbiotics, Lyon, France) and *M. hyo* Ab test (IDEXX Laboratories Inc., Westbrook, ME, USA)]. Testing was conducted in accordance with each manufacturer's kit instructions, where samples were considered as positive for anti‐PCV2 antibodies if the reciprocal ELISA titre was >350 and as positive for *M. hyopneumoniae* antibody if the sample‐to‐positive (S/P) ratio was ≥0.4.

Serum samples were tested for serum virus neutralization using PCV2d strain (SNUVR202002, GenBank no. MW821481) (Fort et al., 2007; Pogranichnyy et al., 2000). Serum samples were heat‐inactivated at 56°C for 30 min prior to performing the test. The neutralization titre with this assay was calculated as the reciprocal of the highest dilution of the serum that was able to 80% block PCV2 infection in PK‐15 cells. Thus, the lowest dilution contained 25% serum (1:1 dilution of serum + equal volume of PCV2d stock), thereby the detection limit of this assay was 2 log_2_.

### 2.10 Enzyme‐linked immunospot

An enzyme‐linked immunospot (ELISpot) assay was conducted to measure the numbers of PCV2d‐ and *M. hyopneumoniae*‐specific interferon‐γ‐secreting cells (IFN‐γ‐SC) (Jeong et al., 2018, 2015). Briefly, 100 ml containing 2 × 10^6^ peripheral blood mononuclear cells (PBMC) in RPMI 1640 medium supplemented with 10% fetal bovine serum (HyClone Laboratories, Inc., SelectScience, Bath, UK) were seeded into plates precoated overnight with anti‐porcine IFN‐γ monoclonal antibody (5 μg/ml, MABTECH, Mariemont, OH, USA) and incubated with PCV2d (20 mg/ml), *M. hyopneumoniae* (4 mg/ml) or phytohemagglutinin (10 mg/ml, Roche Diagnostics GmbH, Mannheim, Germany) as a positive control or PBS as a negative control for 20 h at 37°C in a 5% humidified CO_2_ atmosphere. The wells were washed five times with PBS (200 ml per well) and thereafter, the procedure followed manufacturer's instructions using commercial ELISpot assay kit (MABTECH). The spots on the membranes were read by an automated ELISpot reader (AID ELISpot Reader, AID GmbH, Strassberg, Germany). The results were expressed as the number of responding cells/million PBMC.

### 2.11 Pathology

Two pathologists at the Seoul National University scored the severity of macroscopic lung lesions in order to estimate the percentage of the lung affected by pneumonia (Halbur et al., 1995; Opriessnig et al., 2004). Two blinded veterinary pathologists then examined the collected pulmonary and lymphoid tissue sections. Pulmonary lesions were scored the severity of peribronchiolar lymphoid tissue hyperplasia by mycoplasmal pneumonia lesions ranging from 0 to 6 (0, normal; 1, mild multifocal; 2, mild diffuse; 3, moderate multifocal; 4, moderate diffuse; 5, severe multifocal; 6, severe diffuse) (Opriessnig et al., 2004). Severity of lymphoid lesion severity was scored from 0 to 5 (0, normal; 1, mild lymphoid depletion; 2, mild to moderate lymphoid depletion and histiocytic replacement; 3, moderate diffuse lymphoid depletion and histiocytic replacement; 4, moderate to severe lymphoid depletion and histiocytic replacement; 5, severe lymphoid depletion and histiocytic replacement) (Kim & Chae, 2004b).

### 2.12 Immunohistochemistry

Immunohistochemistry for PCV2 was performed as previously described (Park et al., 2013). Nine sections (three sections from three different blocks) of the same lymph node of each pig were used for the morphometric analyses of immunohistochemistry. Quantitative data were analysed from the prepared immunohistochemistry slides using the NIH Image J 1.45s Program (http://imagej.nih.gov/ij/download.html). PCV2 analysis was conducted by the random selection of 10 microscopic areas, where the number of positive cells per unit area (0.95 mm^2^) was determined as previously described (Kim et al., 2003). The mean values were also calculated.

### 2.13 Statistical analysis

All real‐time PCR data and neutralizing antibody titres were transformed to log_10_ and log_2_, respectively, values prior to statistical analysis. The Shapiro–Wilk test evaluated data for normal distribution. One‐way analysis of variance (ANOVA) was used to examine differences in variables with normal distribution (Average daily weight gain, growth performance, PCV2 DNA, *M. hyopneumoniae* DNA, PCV2 ELISA IgG titre, PCV2 neutralizing antibody titre, *M. hyopneumoniae* ELISA S/P ratio and number of IFN‐γ‐SC). Kruskal–Wallis test was used for variables without a normal distribution (clinical signs, neutralizing antibody titres against PCV2d, macroscopic and microscopic lung lesion scores) for groups. If a one‐way ANOVA test resulted in a statistical significance, data were further evaluated by conducting a post hoc test for a pairwise comparison with Tukey's adjustment. Kruskal–Wallis test results which showed a statistical significance were further evaluated with the Mann–Whitney test to include Tukey's adjustment to compare the differences among the groups. Results were reported in *p*‐value where a value of *p* < 0.05 was considered to be significant.

## 3 RESULTS

### 3.1 Mortality

The overall mortality rate is summarized in Table 2. Diagnostic results indicated that mortality at all farms was primarily related to co‐infection with PCV2 and *M. hyopneumoniae* in unvaccinated animals. Mortality at Farm A was reported as follows: One pig from the VacA1 group died of streptococcal meningitis caused by *Streptococcus suis* at study day 80 (83 days old). Two pigs from the VacA2 group died of streptococcal meningitis caused by *S. suis* at study day 72 (75 days old) and suppurative bronchopneumonia caused by *Staphylococcus aureus* at study day 91 (94 days old), respectively. Two unvaccinated pigs died of enzootic pneumonia caused by *M. hyopneumoniae* and *Pasteurella multocida* at study day 64 and 91 (67 and 94 days old), respectively, and one unvaccinated pig died of PCVAD and Glasser's disease caused by *Glaesserella parasuis* at study day 113 (116 days old).

**TABLE 2:** Body weight (mean ± standard deviation) of pigs vaccinated with trivalent vaccine containing PCV2a/b and *M. hyopneumoniae* or unvaccinated pigs on three swine farms

		Body weight (kg)			
Farm	Group	D 0 (3 days of age)	D 18 (21 days of age)	D 172 (175 days of age)	Mortality
A	VacA1	2.45 ± 0.16	5.83 ± 0.55	106.61 ± 1.54*	5%
	VacA2	2.53 ± 0.21	5.99 ± 0.42	106.06 ± 2.06*	10%
	UnVacA	2.56 ± 0.16	5.84 ± 0.48	100.60 ± 1.73	10%
B	VacB1	2.50 ± 0.24	5.56 ± 0.24	106.21 ± 2.16*	0%
	VacB2	2.50 ± 0.22	5.67 ± 0.22	107.25 ± 1.68*	5%
	UnVacB	2.62 ± 0.16	5.74 ± 0.16	100.28 ± 2.33	15%
C	VacC1	2.57 ± 0.23	5.91 ± 0.52	104.68 ± 1.40*	5%
	VacC2	2.57 ± 0.28	5.82 ± 0.28	104.17 ± 1.95*	0%
	UnVacC	2.72 ± 0.14	5.89 ± 0.43	99.18 ± 1.68	20%

* Significant difference (*p* < 0.05) between vaccinated and unvaccinated group within the same farm.

Mortality at Farm B was reported as follows: One pig from the VacB2 group died of pneumonic pasteurellosis caused by *P. multocida* at study day 57 (60 days old). Two unvaccinated pigs died of severe respiratory disease caused by PCV2d and *M. hyopneumoniae* at study day 82 (85 days old) and enzootic pneumoniae caused by *M. hyopneumoniae* and *P. multocida* at study day 102 (105 days old), respectively. One unvaccinated pig died of lymphoid depletion caused by PCV2d and fibrinous pleuritis and pericarditis caused by *G. parasuis* at study day 93 (96 days old).

Mortality at Farm C was reported as follows: One pig from the VacC1 group died of suppurative bronchopneumonia caused by *S. aureus* at study day 50 (53 days old). Two unvaccinated pigs died of enzootic pneumoniae caused by *M. hyopneumoniae* and *S. aureus* at study day 61 and 68 (64 and 71 days old), respectively. Two additional unvaccinated pigs died of severe respiratory disease caused by PCV2d and *M. hyopneumoniae* at study day 65 and 84 (68 and 87 days old), respectively.

### 3.2 Clinical signs

**FIGURE 1:**
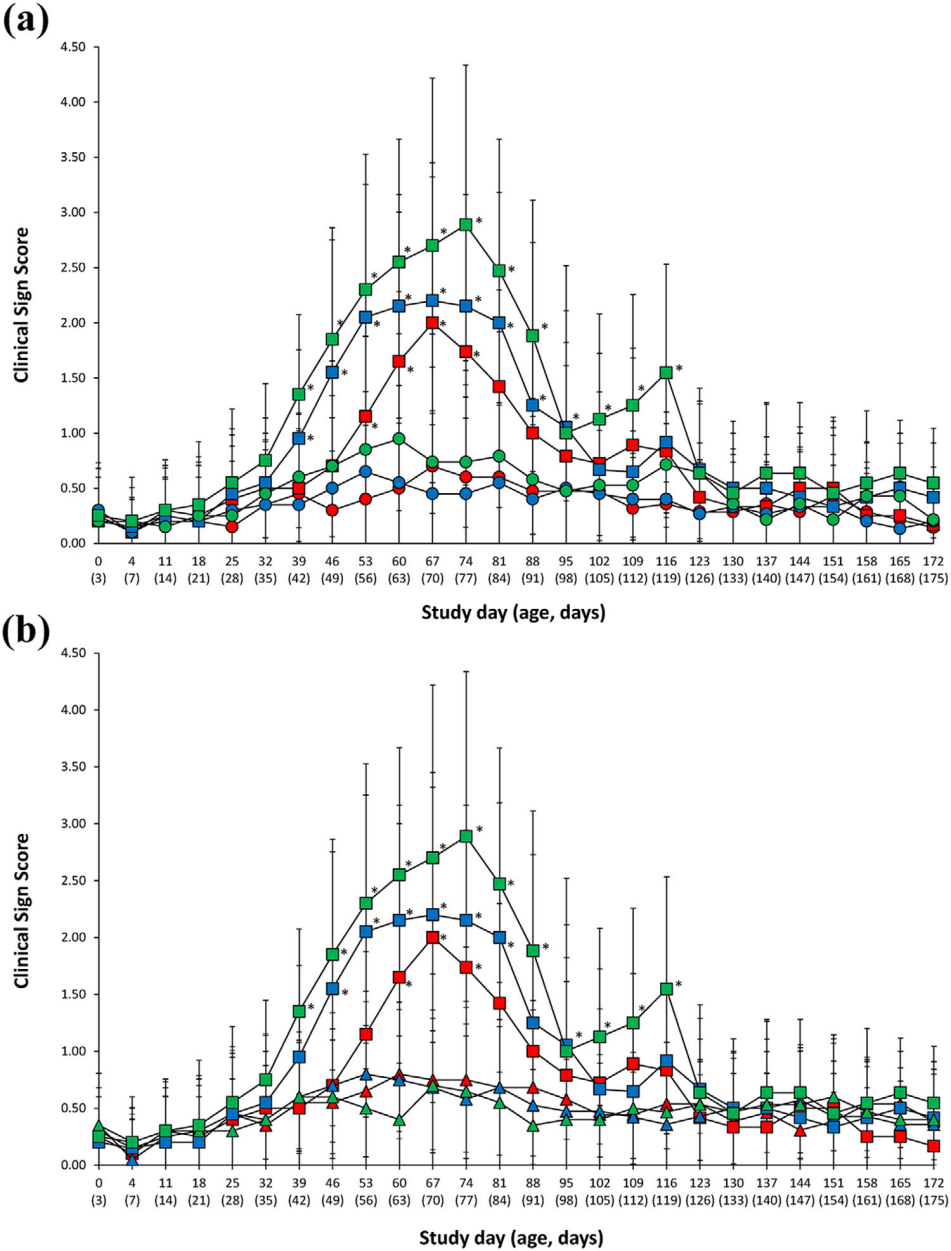
Clinical sign scores. (a) Clinical sign scores (means ± standard deviation) from VacA1 (▴),VacB1 (▴), VacC1 (▴), UnVacA (■), UnVacB (■) and UnVacC (■) groups. (b) Clinical sign scores (means ± standard deviation) from VacA2 (●), VacB2 (●), VacC2 (●), UnVacA (■), UnVacB (■) and UnVacC (■) groups. ^*^Significant difference (*p* < 0.05) between vaccinated and unvaccinated group within the same farm

Vaccinated pigs (VacA1 and VacA2) from farm A had significantly lower (*p* < 0.05) clinical sign scores when compared with unvaccinated animals (UnVacA) at study days 60–74 (Figures 1a and 1b). Farm B vaccinates (VacB1 and VacB2) also had significantly lower (*p* < 0.05) clinical sign scores when compared with unvaccinated animals (UnVacB), but at study days 46–81 (Figures 1a and 1b). On farm C, vaccinated pigs (VacC1 and VacC2) had significantly lower (*p* < 0.05) clinical sign scores when compared with unvaccinated animals (UnVacC) at study days 39–116 (Figures 1a and 1b). A difference in respiratory signs was not observed between one‐dose and two‐dose vaccinated groups in any of the three farms.

### 3.3 Growth performance

The body weight of pigs at study days 0 (3 days of age) and 21 (24 days of age) did not differ significantly between vaccinated and unvaccinated group at the time of vaccination on all three farms. Vaccinated pigs (VacA1, VacA2, VacB1, VacB2, VacC1 and VacC2) had a significantly higher (*p* < 0.05) body weight when compared with unvaccinated pigs in all farms (A–C) at study day 172 (175 day of age) (Table 2).

Vaccinated pigs from all farms (A–C) had significantly higher (*p* < 0.05) ADG at study days 67–109 (70–112 days old) and 109–172 (112–175 days old) when compared with unvaccinated pigs from the same farm. Overall (study days 0–172), the difference between vaccinated and unvaccinated groups was significant (*p* < 0.05) on all farms (Table 3). There were no significant differences in the ADG between one‐dose and two‐dose vaccinated groups on all farms.

**TABLE 3:** Average daily gain (ADG; mean ± standard deviation) in pigs vaccinated with trivalent vaccine containing PCV2a/b and *M. hyopneumoniae* or unvaccinated pigs on three swine farms

		ADG (g/day/pig)			
Farm	Group	D 0–18 (3–21 days old)	D 18–109 (21–112 days old)	D 109–172 (112–175 days old)	D 0–172 (3–175 days old)
A	VacA1	187.50 ± 31.42	588.31 ± 20.01*	794.22 ± 34.13*	605.73 ± 8.45*
	VacA2	192.22 ± 27.07	573.55 ± 18.13*	785.96 ± 33.13*	601.92 ± 11.83*
	UnVacA	182.50 ± 28.34	536.30 ± 22.31	753.70 ± 22.53	569.77 ± 10.01
B	VacB1	169.72 ± 36.41	572.27 ± 18.57*	795.87 ± 33.20*	602.79 ± 12.44*
	VacB2	176.39 ± 19.63	576.26 ± 13.94*	802.04 ± 21.53*	608.76 ± 9.75*
	UnVacB	173.61 ± 25.63	531.02 ± 23.98	746.96 ± 34.62	567.97 ± 13.08
C	VacC1	185.56 ± 32.79	560.94 ± 15.53*	785.15 ± 20.54*	593.90 ± 8.31*
	VacC2	180.83 ± 18.88	556.14 ± 13.45*	785.93 ± 33.31*	590.74 ± 10.57*
	UnVacC	176.11 ± 24.48	518.82 ± 24.11	740.26 ± 35.76	560.73 ± 9.39

* Significant difference (*p* < 0.05) between vaccinated and unvaccinated group within the same farm.

### 3.4 PCV2 viremia

**FIGURE 2:**
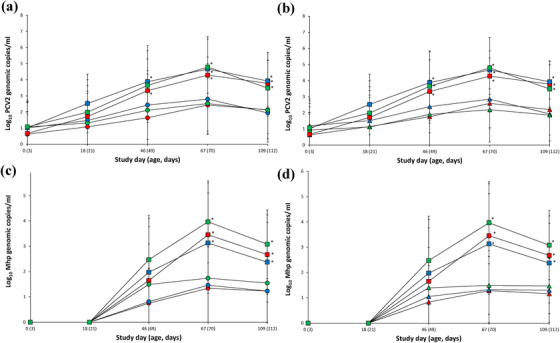
Real‐time PCR results for porcine circovirus type 2d (PCV2d) and *Mycoplasma hyopneumoniae*. (a) Number of PCV2d genomic copies (means ± standard deviation) in the blood from VacA1 (▴),VacB1 (▴), VacC1 (▴), UnVacA (■), UnVacB (■) and UnVacC (■) groups. (b) Number of PCV2d genomic copies (means ± standard deviation) in the blood from VacA2 (●), VacB2 (●), VacC2 (●), UnVacA (■), UnVacB (■) and UnVacC (■) groups. (c) Number of *M. hyopneumoniae* genomic copies in laryngeal swabs from VacA1 (▴), VacB1 (▴), VacC1, (▴), UnVacA (■), UnVacB (■) and UnVacC (■) groups. (d) Number of *M. hyopneumoniae* genomic copies in laryngeal swabs in two‐dose vaccinated (Vac2) and unvaccinated (UnVac) groups. ^*^Significant difference (*p* < 0.05) between vaccinated and unvaccinated group within the same farm

Vaccinated pigs (VacA1, VacA2, VacB1 and VacB2) from farms A and B had a significantly lower (*p* < 0.05) number of genomic copies of PCV2d in their blood when compared with unvaccinated pigs (UnVacA and UnVacB) at study days 46, 67 and 109. Farm C vaccinates (VacC1 and VacC2) also had a significantly lower (*p* < 0.05) number of genomic copies of PCV2d in their blood when compared with unvaccinated pigs (UnVacA and UnVacB) at study days 67 and 109. Two‐dose vaccinated pigs (VacC2) from farm C had a significantly lower (*p* < 0.05) number of genomic copies of PCV2d in their blood when compared with unvaccinated pigs (UnVacC) at study day 46 (Figures 2a and 2b).

The one‐dose and two‐dose vaccinated pigs from farms A, B and C had comparable number of genomic copies of PCV2d DNA throughout the entire field trials with no significant farm‐to‐farm differences between the three sites. Genomic copies of PCV2a and PCV2b DNA were not detected in any pigs from three farms throughout the entire field study.

### 3.5 *Mycoplasma hyopneumoniae* DNA in laryngeal swab

Vaccinated pigs (VacA1, VacA2, VacB1, VacB2, VacC1 and VacC2) from three farms had a significantly lower (*p* < 0.05) number of genomic copies of *M. hyopneumoniae* in their laryngeal swabs when compared with unvaccinated pigs (UnVacA, UnVacB and UnVacC) at study days 67 and 109 (Figures 2c and 2d).

The one‐dose and two‐dose vaccinated pigs from three farms had comparable number of genomic copies of *M. hyopneumoniae* DNA in their laryngeal swabs throughout the entire field trials, and significant differences were not found between groups on the three farms.

### 3.6 Immune responses against PCV2

**FIGURE 3:**
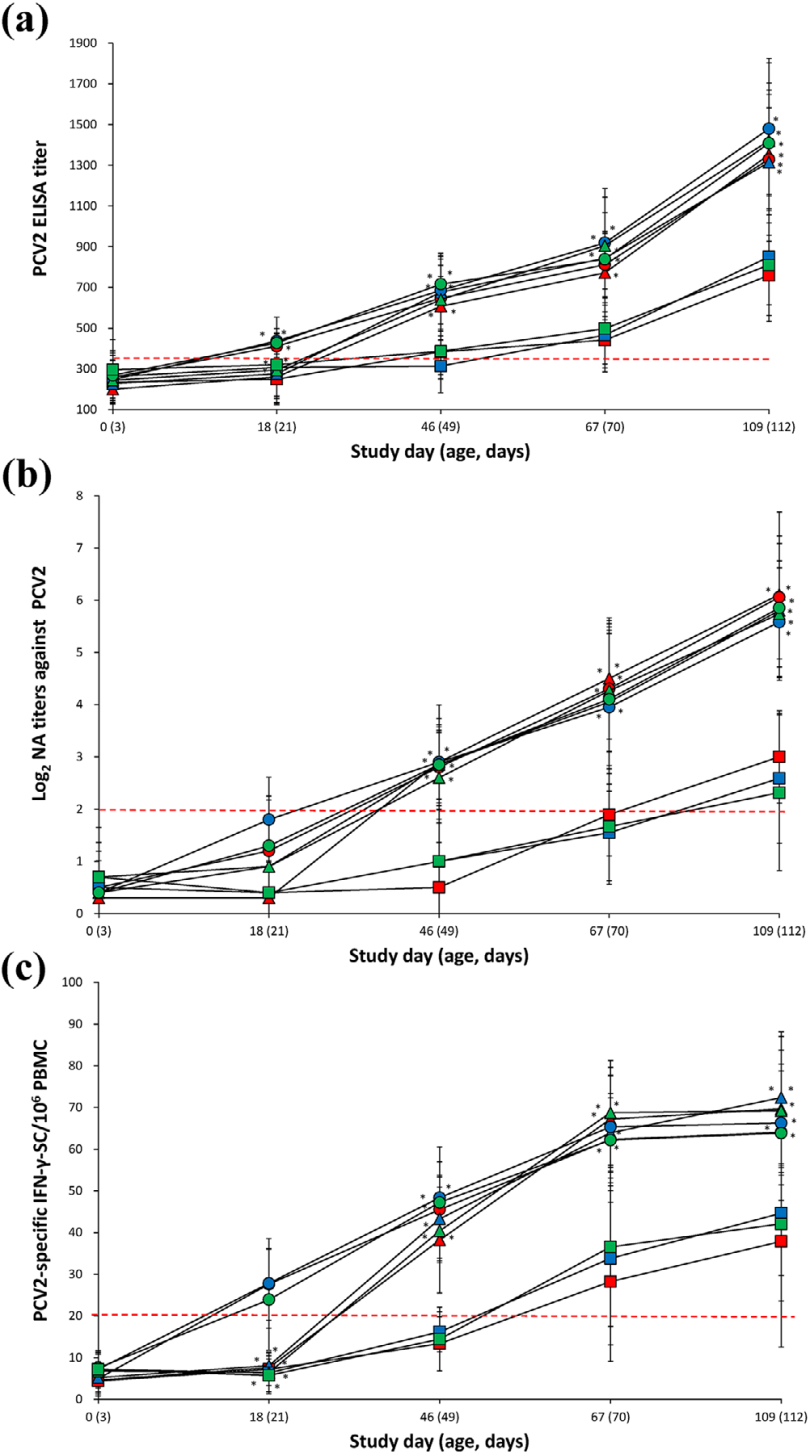
Immune responses against porcine circovirus type 2(PCV2). (a) ELISA titre (means ± standard deviation) in serum samples. (b) Neutralizing antibody (NA) titres against PCV2d in serum samples. (c) ELISpot assay for PCV2d‐specific interferon‐γ‐secreting cells (IFN‐γ‐SC) in peripheral blood mononuclear cells (PBMC) from VacA1 (▴), VacB1 (▴), VacC1 (▴), VacA2 (●), VacB2 (●), VacC2 (●), UnVacA (■), UnVacB (■) and UnVacC (■) groups. Red dotted line is cuff‐off (ELISA titre >350, NA titre >2 log_2_ and ELISpot number of PCV2d‐specific IFN‐γ‐SC >20 cells/10^6^ PBMC). ^*^Significant difference (*p* < 0.05) between vaccinated and unvaccinated group within the same farm

Two‐dose vaccinated pigs (VacA2, VacB2 and VacC2) from three farms had a significantly higher (*p* < 0.05) PCV2 ELISA IgG titre at study day 18 when compared with one‐dose vaccinated (VacA1, VacB1 and VacC1) and unvaccinated (UnVacA, UnVacB and UnVacC) pigs. Vaccinated pigs (VacA1, VacA2, VacB1, VacB2, VacC1 and VacC2) from three farms had a significantly higher (*p* < 0.05) PCV2 ELISA IgG titre at study days 46, 67 and 109 when compared with unvaccinated pigs (Figure 3a). Vaccinated pigs (VacA1, VacA2, VacB1, VacB2, VacC1 and VacC2) from three farms had a significantly higher (*p* < 0.05) PCV2 neutralizing antibody titre at study days 46, 67 and 109 when compared with unvaccinated pigs (Figure 3b).

Two‐dose vaccinated animals (VacA2, VacB2 and VacC2) from three farms had a significantly higher (*p* < 0.05) number of PCV2d‐specific IFN‐γ‐SC at study day 18 when compared with one‐dose vaccinated (VacA1, VacB1 and VacC1) and unvaccinated (UnVacA, UnVacB and UnVacC) pigs. Vaccinated pigs (VacA1, VacA2, VacB1, VacB2, VacC1 and VacC2) from three farms had a significantly higher (*p* < 0.05) number of PCV2d‐specific IFN‐γ‐SC at study days 46, 67 and 109 when compared with unvaccinated pigs (Figure 3c).

### 3.7 Immune responses against *M. hyopneumoniae*

**FIGURE 4:**
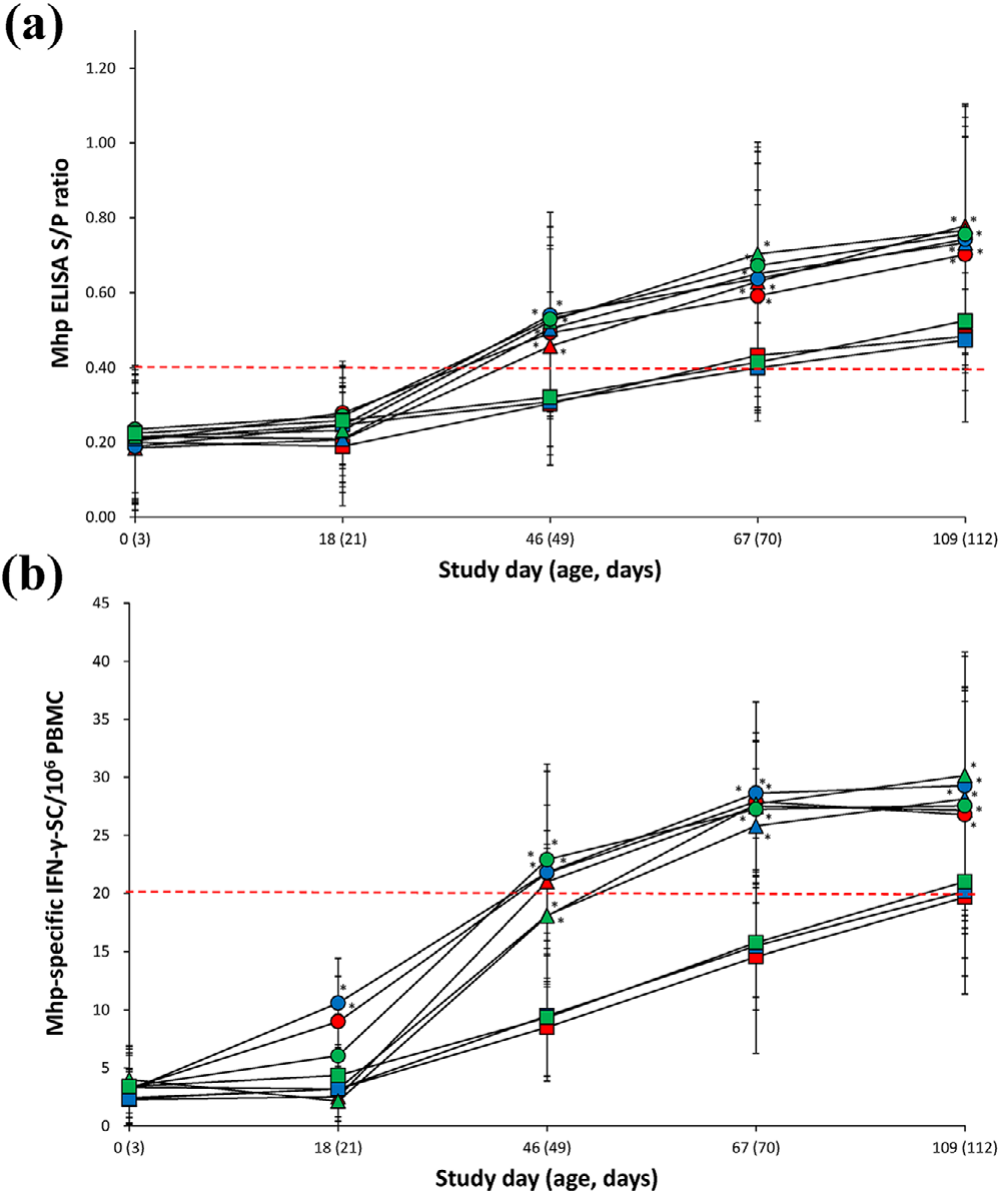
Immune responses against *Mycoplasma hyopneumoniae*. (a) ELISA sample‐to‐positive (S/P) ratio (means ± standard deviation) in serum samples. (b) ELISpot assay for *M. hyopneumoniae* (Mhp)‐specific interferon‐γ‐secreting cells (IFN‐γ‐SC) in peripheral blood mononuclear cells (PBMC) from VacA1 (▴), VacB1 (▴), VacC1 (▴), VacA2 (●), VacB2 (●), VacC2 (●), UnVacA (■), UnVacB (■) and UnVacC (■) groups. Red dotted line is cuff‐off (ELISA S/P ratio ≥0.4 and ELISpot number of *M. hyopneumoniae*‐specific IFN‐γ‐SC >20 cells/10^6^ PBMC). ^*^Significant difference (*p* < 0.05) between vaccinated and unvaccinated group within the same farm

Vaccinated pigs (VacA1, VacA2, VacB1, VacB2, VacC1 and VacC2) from three farms had a significantly higher (*p* < 0.05) *M. hyopneumoniae* ELISA S:P ratio at study days 46, 67 and 109 when compared with unvaccinated pigs (UnVacA, UnVacC and UnVacC) (Figure 4a).

Two‐dose vaccinated pigs (VacA2 and VacB2) from farms A and B had a significantly higher (*p* < 0.05) number of *M. hyopneumoniae*‐specific IFN‐γ‐SC at study day 18 when compared with the unvaccinated pigs. Vaccinated pigs (VacA1, VacA2, VacB1, VacB2, VacC1 and VacC2) from three farms had a significantly higher (*p* < 0.05) number of *M. hyopneumoniae*‐specific IFN‐γ‐SC at study days 46 and 67 when compared with unvaccinated pigs (UnVacA, UnVacC and UnVacC) (Figure 4b).

### 3.8 Pathology

Mycoplasmal lung lesions were characterized by various degree of peribronchiolar lymphoid tissue hyperplasia. PCV2‐associated lesions in lymphoid tissues were characterized by lymphoid depletion and histiocytic‐to‐granulomatous inflammation with/without low‐to‐moderate numbers of multinucleated giant cells. Vaccinated pigs (VacA1, VacA2, VacB1, VacB2, VacC1 and VacC2) from three farms had significantly lower (*p* < 0.05) macroscopic lung lesion score, microscopic lung and lymphoid lesion scores and number of lymphoid PCV2‐positive cells when compared to unvaccinated pigs (UnVacA, UnVacB and UnVacC) at study day 109 (Tables 4 and 5). On farm C, vaccinated pigs (VacC1 and VacC2) had significantly lower (*p* < 0.05) microscopic lung and lymphoid lesion scores, and number of lymphoid PCV2‐positive cells when compared to unvaccinated pigs (UnVacA, UnVacB and UnVacC) at study day 172 (Tables 4 and 5). There were no significant differences in overall scores for microscopic lung and lymphoid lesions, and the numbers of lymphoid PCV2‐positive cells between one‐dose and two‐dose vaccination regimens.

**TABLE 4:** Lung lesion scores (means ± standard deviation)

		Macroscopic lesions		Microscopic lesions	
Farm	Group	D 109 (112 days old)	D 172 (175 days old)	D 109 (112 days old)	D 172 (175 days old)
A	VacA1	22.5 ± 9.8*	17.8 ± 1.7	0.80 ± 0.18*	0.52 ± 0.30
	VacA2	24.8 ± 9.3*	17.3 ± 3.3	0.88 ± 0.24 *	0.72 ± 0.16
	UnVacA	47.2 ± 11.0	25.6 ± 10.4	2.64 ± 0.56	0.96 ± 0.45
B	VacB1	24.7 ± 5.5*	18.9 ± 5.9	1.00 ± 0.33*	0.80 ± 0.25
	VacB2	25.2 ± 4.1*	18.8 ± 6.3	1.12 ± 0.50*	0.72 ± 0.16
	UnVacB	45.4 ± 10.5	27.1 ± 6.4	2.40 ± 0.44	1.12 ± 0.52
C	VacC1	26.4 ± 3.8*	22.3 ± 8.0*	1.28 ± 0.27*	0.96 ± 0.08*
	VacC2	25.5 ± 7.9*	23.7 ± 5.7*	1.08 ± 0.56*	0.92 ± 0.32*
	UnVacC	52.1 ± 6.4	34.6 ± 4.3*	3.80 ± 0.28	2.12 ± 0.37

* Significant difference (*p* < 0.05) between vaccinated and unvaccinated group within the same farm.

**TABLE 5:** Lymphoid lesion scores and PCV2‐positive cells (means ± standard deviation)

		Microscopic lesions		No. of PCV2‐positive cells	
Farm	Group	D 109 (112 days old)	D 172 (175 days old)	D 109 (112 days old)	D 172 (175 days old)
A	VacA1	0.96 ± 0.29*	0.64 ± 0.20	4.33 ± 0.63*	2.80 ± 0.91
	VacA2	0.84 ± 0.39*	0.76 ± 0.37	4.93 ± 0.90*	3.13 ± 1.05
	UnVacA	2.04 ± 0.41	1.04 ± 0.43	10.87 ± 1.24	4.07 ± 1.08
B	VacB1	1.04 ± 0.20*	0.60 ± 0.33	5.33 ± 0.76*	3.13 ± 0.75
	VacB2	0.92 ± 0.32*	0.56 ± 0.27	5.80 ± 0.34*	3.47 ± 1.05
	UnVacB	2.20 ± 0.36	1.00 ± 0.42	11.60 ± 2.00	4.80 ± 0.86
C	VacC1	1.16 ± 0.32*	0.92 ± 0.20*	5.93 ± 0.33*	3.60 ± 0.93*
	VacC2	1.12 ± 0.20*	0.96 ± 0.08*	6.20 ± 0.50*	3.73 ± 0.98*
	UnVacC	3.04 ± 0.46	1.40 ± 0.28	14.07 ± 1.39	5.93 ± 1.18

* Significant difference (*p* < 0.05) between vaccinated and unvaccinated group within the same farm.

## 4 DISCUSSION

The common sign of PCV2 and *M. hyopneumoniae* co‐infection is growth retardation. Vaccination against these two pathogens is needed and widely used to improve pig growth performance. Therefore, growth performance was selected as the most critical index in the efficacy evaluation of a trivalent vaccine under field conditions. The pigs vaccinated with the trivalent vaccine demonstrated improved growth performance suggesting that the vaccine may have contributed to the favourable outcome in the farm A and B herds with subclinical PCV2 infection. Overt clinical signs of PCVAD were not observed on either of these two farms. Such field observations have also been reported in other pig rearing countries such as Canada (Young et al., 2011), the United Kingdom (Alarcon et al., 2013b), Spain (Fraile et al., 2012), Germany (Heißenberger et al., 2013) and Switzerland (Kurmann et al., 2011). Swine practitioners and producers are therefore aware of the costly impact that subclinical PCV2 infection has in swine herds. This may directly impact the decision of producers to vaccinate animals even in the absence of overt clinical signs of PCVAD.

The trivalent vaccine containing PCV2a/b and *M. hyopneumoniae* evaluated in the field trials elicited protective immunity against PCV2d and *M. hyopneumoniae*. Protective immunity in the forms of PCV2‐specific neutralizing antibodies and IFN‐γ‐SC reduced the amount of PCV2 viral blood load and reduced the severity of lymphoid lesions (Fort et al., 2008, 2009; Meerts et al., 2006, 2005). For the aspect of immune responses to *M. hyopneumoniae*, humoral immunity has not been associated with protection (Djordjevic et al., 1997) but cell‐mediated immunity plays a role to protect pigs from *M. hyopneumoniae* infection (Thacker et al., 2000). Trivalent vaccine was successful in inducing a measurable cellular immune response and reducing the severity of mycoplasmal lung lesions. Significant differences were not observed between the one‐dose and two‐dose vaccinated groups in relation to the induction of detectable immune response against PCV2 and *M. hyopneumoniae*, the reduction of genomic copies of PCV2 in blood and *M. hyopneumoniae* in laryngeal swabs and the reduction of pulmonary and lymphoid lesion severity. The trivalent vaccine administered as either one or two doses therefore elicited detectable immune response and provided protection against PCV2 and *M. hyopneumoniae* infection.

Pathological evaluation was also critical in evaluating the protective index as lesion reduction is related to growth performance in both PCV2 and *M. hyopneumoniae* infection (Jensen et al., 2002; Maes et al., 1998; Martelli et al., 2011; Segalés et al., 2009). No differences in lymphoid lesions or lymphoid PCV2 antigen‐positive cells were observed between vaccinated and unvaccinated animals at study day 172 (175 days old) in the two farms (farms A and B) with subclinical PCV2 infection. The minimal or mild lymphoid lesion severity and low number of PCV2 antigen‐positive cells of farm A and B pigs were consistent with the definition of subclinical infection (Segalés, 2012). Unlike the two farms (A and B) with a history of subclinical PCV2, on farm C with a history of PCVAD statistical differences in lymphoid lesions and lymphoid PCV2‐positive cells were observed between vaccinated and unvaccinated animals at study day 172 (175 days old). Mycoplasmal pneumonic lesions and laryngeal swab load from pigs at study day 109 (112 days old) were significantly reduced in the vaccinated group when compared to the unvaccinated group in all three farms. Mycoplasmal pneumonia resolved in finishing pigs by study day 172 (175 days old) in farms A and B (which had subclinical PCV2 infection and enzootic pneumonia). Co‐infection of pigs with PCV2 and *M. hyopneumoniae* causes PRDC and exacerbates lung lesion severity. This was observed in the Farm C finishing pigs at study day 172 (175 days old), where the effect of vaccination on the reduction of lung lesion severity was proven. Commercial farm pigs used as field trials such as this are continuously exposed and re‐exposed to the prevalent field PCV2d and *M. hyopneumoniae* by horizontal transmission. Natural co‐infections as well as other intrinsic and extrinsic factors also exacerbate disease in these less‐controlled commercial settings. A true evaluation of the direct effect of vaccination on pathological outcomes would require a controlled experimental challenge study.

Piglets also face potential interference from maternally derived antibodies (MDA) present at the time of vaccination. In general, early vaccination against PCV2 and *M. hyopneumoniae* was proven as effective in piglets less than 1 week of age regardless of MDA presence (O'Neill et al., 2011; Wilson et al., 2012). This field study did not evaluate the effect of MDA on vaccine efficacy. Additional studies are necessary to explore this theory and ultimately determine the effect of MDA on trivalent vaccine efficacy under well‐controlled experimental conditions.

## CONFLICT OF INTEREST

The authors declare no conflict of interest.

## AUTHOR CONTRIBUTIONS

Hyungmin Um conceptualized the idea of the study, curated the data, performed investigation and designed the methodology. Siyeon Yang curated the data, provided resources and performed visualization. Taehwan Oh designed the methodology and provided software. Hyejean Cho performed formal analysis. Kee Hwan Park performed validation. Jeongmin Suh provided software. Chanhee Chae conceptualized the idea of the study, administered the project, performed supervision and reviewed and edited the manuscript.

## ETHICAL APPROVAL

All of the methods were previously approved by the Seoul National University Institutional Animal Care and Use, and Ethics Committee (approval number SNU‐191017‐10). Sample collection was carried out according to the animal welfare code of Korea.

5

### PEER REVIEW

The peer review history for this article is available at https://publons.com/publon/10.1002/vms3.657.

## Data Availability

The data that support the findings of this study are available from the corresponding author upon reasonable request.
